# Mitral Annuloplasty Using a Cardiac Resynchronization Device

**Published:** 2010-07-20

**Authors:** Andrabi Syed Manzoor Ali, Khurshid Iqbal, Nisar Ahmed Trambu

**Affiliations:** Department of Cardiology, SKIMS, Soura, Srinagar 190010, India.

**Keywords:** Mitral annuloplasty, Cardiac Resynchronization Device

## Abstract

Percutaneous Transvenous Mitral Annuloplasty for mitral regurgitation is in early stages of development and involves a complex intervention which can not be done in patients with left ventricular leads. Since functional mitral regurgitation is common in low ejection fraction states, we propose a device which can serve for annuloplasty in addition to cardiac resynchronization therapy and simplifying the intervention.

Treatment of dilated cardiomyopathy has evolved considerably over years leading to marked improvement in quality of life and survival. This has been possible by intervening at the various biochemical, hemodynamic and electrical abnormalities by drugs and  use of  various devices. The role of cardiac resynchronization therapy (CRT) is an established one resulting in improvement in quality of life and survival and lately studies have shown  its benefit even in patients with  moderate fall in ejection fraction.

One important accompaniment of dilated heart is functional mitral regurgitation secondary to mitral annular dilatation, apical displacement of papillary muscles and reduced transmitral closure force  secondary to LV systolic dysfunction.

Cardiac resynchronization therapy helps by synchronizing the contractions of the postero-lateral wall and the interventricular septum and thereby improving the ejection fraction and by decreasing the functional mitral regurgitation by its presumed effect of synchronizing the motion of two papillary muscles and increasing transmitral closure force.

Percutaneous  transvenous mitral  annuloplasty (PTMA) for ischemic or functional mitral regurgitation is in its evolution. The procedure involves cannulation of coronary sinus via subclavian or internal jugular  vein and delivering  a hemispherical  structure from coronary sinus ostium posteriorly to the anerior interventricular branch of the  great cardiac vein anteriorly. As the coronary sinus  forms  the lateral half of the mitral annulus, the device alters  the geometry of the mitral annulus  thereby causing better apposition of two valve cusps  and decreasing mitral regurgitation. Three such devices under investigation include Viacor PTMA device, CARILLON contour system and MONARC (previously VIKING) PTMA system all utilizing the various modifications of the same principle. These PTMA devices cannot be placed in patients who have coronary sinus leads of CRT devices. Since low ejection fraction states are usually associated with functional mitral regurgitation hence to produce a device which can address both issues seems compelling.

Although the left ventricular leads of CRT devices are soft and may not significantly bring about conformational changes in the mitral annulus, part of reduction of mitral regurgitation can be expected by left ventricular lead especially once the lead is distally nicely fixed and under some tension. However  left ventricular lead is placed in the  lateral most  vein  which may include  only one fourth of the mitral annulus. If  left ventricular lead is modified in such a way that it is rendered  stiff in its distal part and part of the stiff end projects anteriorly upto anterior interventricular groove  it can serve as well for annuloplasty in addition to CRT. Otherwise a stiff  annuloplasty hemisphere can be delivered over the left ventricular lead hence simplifying the procedure and simultaneously addressing both issues.

## Figures and Tables

**Figure 1:**
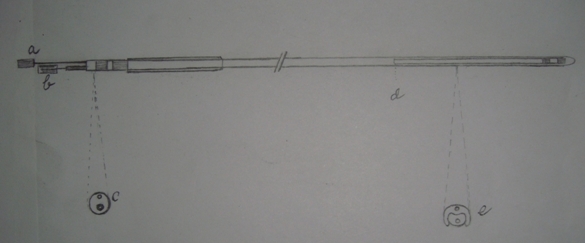
Proposed  left ventricular lead  bifurcating distally at 'd' into outer jacket  for annuloplasty  and core lead for CRT both having  separate stylets 'a' and 'b' for manipulation.'C' and  'e' show the cross sections  of lead at proximal and distal  ends after  bifurcation.

**Figure 2:**
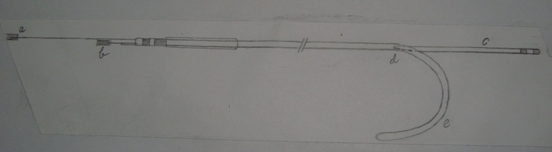
After withdrawing  the stylet  'a' out of the annuloplasty lead, its distal end 'e'  takes predefined hemispherical shape which will help in scaffolding of mitral annulus improving  mitral leaflet coaptation  while 'c' the CRT lead is positioned in one of the lateral cardiac veins.

**Figure 3:**
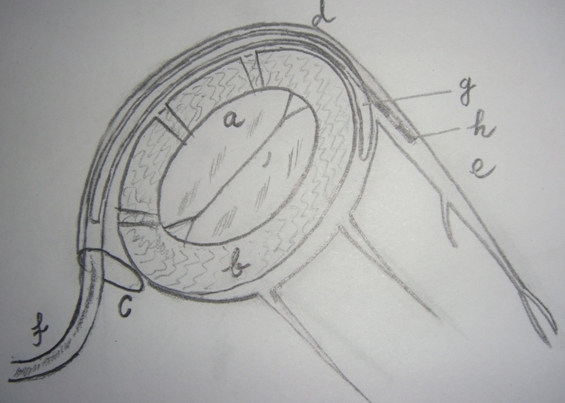
After reaching the desired lateral cardiac vein 'e' in the coronary sinus 'd'  the CRT lead  'h' is directed across its ostium  to the tip while the annuloplasty jacket  lead  is pushed distally as far as possible  in the coronary sinus surrounding mitral valve annulus 'b'. 'c' coronary sinus ostium.'f'  left ventricular lead.

**Figure 4:**
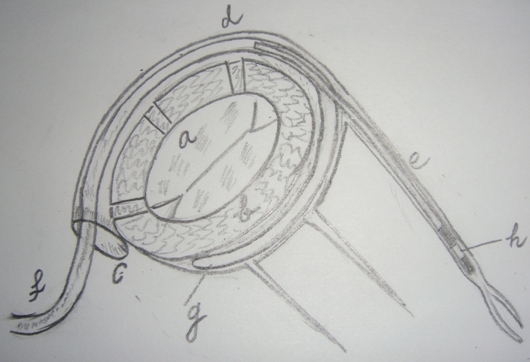
Final position of the bifurcated lead with annuloplasty part 'g' in the coronary sinus surrounding major part of mitral valve 'a' annulus 'b' and the CRT lead 'h' in the desired lateral  cardiac vein 'e'.The distal annuloplasty segment can be made of material that utilizes a slow conformational change because of delayed breakdown of biodegradeable polymer over 3 to 6 weeks resulting in further narrowing of mitral annulus.

